# Impaired synaptic transmission in dorsal dentate gyrus increases impulsive alcohol seeking

**DOI:** 10.1038/s41386-022-01464-5

**Published:** 2022-10-01

**Authors:** Maria Nalberczak-Skóra, Anna Beroun, Edyta Skonieczna, Anna Cały, Magdalena Ziółkowska, Roberto Pagano, Pegah Taheri, Katarzyna Kalita, Ahmad Salamian, Kasia Radwanska

**Affiliations:** 1 Laboratory of Molecular Basis of Behavior, Nencki Institute of Experimental Biology of Polish Academy of Sciences, Warsaw, Poland; 2 Experimental Psychopathology Lab, Institute of Psychology of Polish Academy of Sciences, Warsaw, Poland; 3 BRAINCITY, Nencki Institute of Experimental Biology of Polish Academy of Sciences, Warsaw, Poland

**Keywords:** Addiction, Cellular neuroscience

## Abstract

Both human and animal studies indicate that the dentate gyrus (DG) of the hippocampus is highly exploited by drug and alcohol abuse. Yet, it is poorly understood how DG dysfunction affects addiction-related behaviors. Here, we used an animal model of alcohol use disorder (AUD) in automated IntelliCages and performed local genetic manipulation to investigate how synaptic transmission in the dorsal DG (dDG) affects alcohol-related behaviors. We show that a cue light induces potentiation-like plasticity of dDG synapses in alcohol-naive mice. This process is impaired in mice trained to drink alcohol. Acamprosate (ACA), a drug that reduces alcohol relapse, rescues the impairment of dDG synaptic transmission in alcohol mice. A molecular manipulation that reduces dDG synaptic AMPAR and NMDAR levels increases impulsive alcohol seeking during cue relapse (CR) in alcohol mice but does not affect alcohol reward, motivation or craving. These findings suggest that hindered dDG synaptic transmission specifically underlies impulsive alcohol seeking induced by alcohol cues, a core symptom of AUD.

## Introduction

Alcohol use disorder (AUD), commonly named alcohol addiction, is a chronic psychiatric disease characterized by a high probability of relapse to excessive alcohol use even after protracted abstinence [1]. Both human and animal studies indicate that alcohol craving, and consequent relapse to alcohol drinking, can be triggered by alcohol cues, e.g., environmental stimuli that are associated with alcohol consumption or predict alcohol availability [2–4]. Thus, understanding the neuronal processes that control cue relapse (CR) is required to facilitate the development of pharmacotherapies to treat addiction.

Reinstatement of alcohol and drug seeking induced by environmental cues has been linked with aberrant activity of the prefrontal cortex (PFC), nucleus accumbens (NAc), amygdala, and hippocampus [4–12]. For example, upon presentation of cocaine-associated cues, the excitatory synapses on medium spiny neurons in NAc are potentiated, as indicated by increased dendritic spine head diameter, elevated AMPA receptor-mediated synaptic currents and AMPA receptor surface expression [5, 13, 14]. These synaptic changes are suggested to mediate the enhanced motivation underlying relapse to drug use [7]. The synaptic mechanisms that govern other aspects of CR (e.g., control of impulsive responses or representation of cue value) likely engage other neuronal pathways of the CR circuits, but they remain less understood. It is also unknown how the synaptic plasticity induced by drug cues differs from the synaptic processes induced by neutral stimuli.

The dentate gyrus (DG) of the hippocampus is highly exploited in alcohol and drug abuse. Chronic alcohol consumption increases cell death and decreases adult neurogenesis, cell proliferation [15–17], and the overall number of DG granule cells both in rats [18, 19] and human patients diagnosed with AUD [20]. Manipulations that ablate adult neurogenesis in the DG increase drug consumption and motivation to seek drugs, as well as invigorate reinstatement of drug seeking induced by associated cues and contexts [21, 22], suggesting that impaired DG connectivity contributes to the addiction-like phenotype. In agreement with this hypothesis, our previous data showed that the presentation of alcohol-predicting cues to the animals trained to drink alcohol results in the generation of silent glutamatergic synapses (lacking functional AMPAR) in the dorsal DG (dDG). This phenomenon is more pronounced in animals with an addiction-like phenotype, as compared to addiction-resistant ones, and it is absent in animals responding to cues associated with water or sucrose reward [11]. Still, how the dDG processes alcohol-predicting cues remain largely unknown. The goals of this study was to characterize the molecular, structural and functional changes induced in the dDG circuit by alcohol-predicting cues, to determine if they differ from the processes induced by neutral sensory cues, to validate if the dDG regulates alcohol seeking, and finally, the effects of acamprosate (ACA) treatment on alcohol mice as this drug significantly decreases alcohol relapse and consumption in human alcoholics [23, 24].

To test the role of dDG synaptic plasticity in the regulation of alcohol seeking induced by alcohol-predicting cues, we used a comprehensive mouse model of AUD in our automated IntelliCages setup [25]. The IntelliCage allowed us to not only observe the animals’ behavior during the presentation of alcohol-predicting cues but to also assess alcohol reward, motivation to drink alcohol, persistence in alcohol seeking during protracted abstinence and under conditions when alcohol availability is temporarily limited. We used diolistic labeling [26] to analyze the morphology of dendritic spines (as a proxy of glutamatergic synapses) in dDG, and whole-cell patch-clamp electrophysiology to analyze the ratio of AMPA and NMDA currents, as a measure of synaptic strength [27]. Overall, our data indicate that the synaptic strengthening induced in the dDG by the neutral light cue in alcohol-naïve mice is impaired in the alcohol mice exposed to the alcohol-predicting cue, and this is rescued by ACA treatment. Reduced dDG synaptic transmission in alcohol mice specifically enhances impulsive alcohol seeking during CR, and does not affect alcohol reward, motivation to drink, alcohol craving during extinction or persistence in alcohol seeking.

## Materials and methods

A detailed description of all procedures is in the Supplementary Section.

### Animals

Eight-week-old, C57BL/6J mice were provided by the Medical University of Białystok, Poland. Mice had ad libitum access to water and food before and during the experiments. Due to the high aggression of the males group-housed in the IntelliCages, all experiments were conducted only on the females. All biochemical and electrophysiological analyses were performed after the training in the IntelliCages when the animals were 19–20-week old. The experiments were designed to minimize the number of animals and their suffering. All experiments were approved by the 1st Local Ethical Committee in Warsaw, Poland (approvals: 421/2017 and 711/2018) and were conducted in agreement with the Animal Protection Act of Poland.

### Animal model of alcohol addiction

Fourteen to sixteen mice were trained per one IntelliCage and they were simultaneously tested for AUD-related behaviors as previously described [11, 25]. The timelines of the experiments are shown in the figures and described in Supplementary Materials.

### Acamprosate treatment

After long-term ethanol (or water) self-administration, mice were treated with acamprosate (ACA) (0.04% solution of *Campral* (Merck) in tap water). Mice had ad libitum access to the bottles with water or alcohol only after they drank 500 licks of ACA solution, leading to 250 mg/kg/day consumption, which is known to reduce alcohol drinking in rats and mice [11, 28].

### Western blot

The membranes were incubated with the primary antibody (GluA1 1:1000, Abcam #109450; GluA2 1:5000, Abcam #133477; GluN1 1:1000, Sigma #G8913; GluN2A 1:2000, Merck/Millipore #05-901R; GluN2B 1:3000, Abcam #183942; GluN3A 1:1000, Merck/Millipore #07-356), followed by a secondary antibody with HRP (1:5000, Vector pI-1000). TGX stain-free gels from Bio-Rad (#4568095) were used to standardize protein loading on gels [29].

### Immunofluorescent staining

The brain sections were incubated with primary antibody (PSD-95, Millipore, #MAB1598, 1:500; GFP, Synaptic Systems #132004; 1:2000), followed by secondary antibodies (1:500; Invitrogen, Alexa Fluor 488, #1182671 and 555, #1736967). The fluorescent staining in the upper blade of dDG [30] was photographed with a confocal microscope (Zeiss LSM800, magnification ×63).

### DioIistic staining

Six slices containing dorsal hippocampus per animal were labeled by tungsten particles (Bio-Rad, #156-2268) coated with the lipophilic dye (1,1-dioctadecyl-3,3,3,3-tetramethylindocarbocyanine perchlorate, DiI; Life Technologies, D-282). Z-stacks of confocal images of the 5–7 dendrites per animal from the middle molecular layer of the upper blade of dDG were acquired using Zeiss Spinning Disc confocal microscope (lens: 63× oil immersion, pixel size: 132 × 132 × 260 nm). Neurons of immature morphology (fewer dendrites, very sparse dendritic spines) were excluded from the analysis. Spine clustering into thin, stubby and mushroom was automatically performed using NeuronStudio software.

### Statistical analysis

The sample sizes of the experimental groups, and details of the statistics, are placed on the graphs or in the legends. The data with normal distribution and equal variance are presented as the mean with SEM and were analyzed with Student’s *t* test, one-way or two-way ANOVA. Post hoc Sidak’s and Tukey’s tests for multiple comparisons were used. Alternatively, two-way repeated-measure (RM) ANOVA with two-stage linear step-up procedure of Benjamini, Krieger, and Yekutieli for multiple comparisons. The difference between the experimental groups was considered significant at *p* < 0.05. All statistical analyses were performed using GraphPad Prism 9.3.1 Software.

## Results

### Behavioral analysis of alcohol and alcohol-naive mice in the IntelliCages

**Fig. 1:**
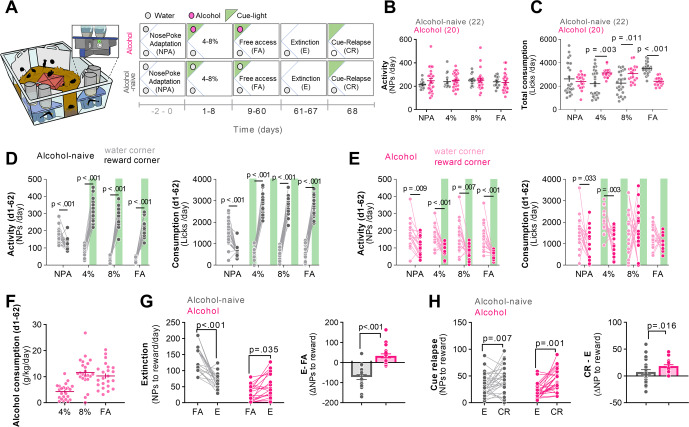
Cue relapse in the IntelliCages is enhanced in alcohol mice, as compared to alcohol-naive animals. **A** Diagram of IntelliCage setup with a magnified corner and experimental timeline. Phases of the training: nose-poke adaptation (NPA), initiation of alcohol consumption (4–8%) followed by free alcohol access (FA), extinction (E), and cue relapse (CR). During alcohol FA both cage corners were active and a green cue light predicting alcohol (or water) was presented each time a mouse entered the reward corner. During E the reward corner was inactive and mice had no access to alcohol (or water) in this corner. During CR the cue light was on each time a mouse entered the reward corner but no alcohol (or water) was available. **B–F** Summary of data showing mice activity during periods of FA to alcohol: (**B**, **C**) total consumption (repeated measures two-way ANOVA with Šídák’s multiple comparisons test; alcohol × time interaction: *F* (4, 164) = 23.3, *p* < 0.001) and nose-pokes (NPs) (repeated measures two-way ANOVA, alcohol: *F* (1, 39) = 0.557, *p* = 0.460); (**D**, **E**) consumption (repeated-measure two-way ANOVA with Šídák’s multiple comparisons test, alcohol-naive, effect of corner: *F* (1,00, 26,0) = 262, *p* < 0.001; alcohol group, effect of corner: *F* (1,00, 17,0) = 38.5, *p* < 0.001) and NPs in two cage corners (repeated-measure two-way ANOVA with Šídák’s multiple comparisons test, alcohol-naive, effect of corner: *F* (1,00, 26,0) = 567, *p* < 0.001; alcohol group, effect of corner: *F* (1,00, 17,0) = 8.98, *p* = 0.008); (**F**) alcohol consumption. **G** Summary of data showing reward seeking during W. (*left*) Mice activity in the reward corner during W, as compared to the last day before the test (two-way ANOVA, training × alcohol: *F* (1, 80) = 37.73, *p* < 0.001; Sidak’s post hoc test for alcohol: *p* = 0.011; alcohol-naive: *p* < 0.001) and (*right*) the change of activity during E (*t* test, t(40) = 6.75, *p* < 0.001). **H** Summary of data showing reward seeking during CR. (*left*) Mice activity in the reward corner during CR, as compared to the last day before the test (training: *F* (1, 106) = 17.95, *p* < 0.0001, alcohol: *F* (1, 106) = 3.332, *p* = 0.070; Sidak’s post hoc test for alcohol-naive: *p* = 0.007, alcohol: *p* = 0.001). (*right*) The change of activity during CR (Mann–Whitney *U* test = 125.5, *p* = 0.016). *p < 0.05; **p < 0.01; ***p < 0.001 by Tukey’s post hoc tests. Numbers of animals in the experimental groups are shown on the graphs (each dot represents one animal).

Mice were trained to drink ethyl alcohol in IntelliCages based on our previously published protocols [11, 25]. They had unlimited access to water throughout the whole experiment in one cage corner and to alcohol (Alcohol group, A) or water (Alcohol-naive group, An) in the reward corner according to the schedule. The training was composed of the following phases: nose-poke adaptation (NPA, days −2–0, water in both corners), introduction of alcohol in increasing concentrations (4 and 8%, days 1–8; reward corner active: each visit activated a cue light in the corner, each nose-poke (NPs) gave access to alcohol for 5 s), free access to 12% alcohol (FA, days 9–60, reward corner active), extinction (E, days 61–67; reward corner inactive: visits and NPs had no programed consequences), and cue relapse (CR, days 68; each visit activated the cue light in the reward corner but alcohol (or water) was not available) (Fig. 1A).

Mice in the two experimental groups did not differ in general activity (measured as NPs in two cage corners) (Fig. 1B), however, during 4–8% phases the alcohol mice drank more compared to alcohol-naive animals (Fig. 1C). The cue light had rewarding properties for the alcohol-naive mice (Fig. 1D) as indicated by their preference to the reward corner (both NPs and licks). On the other hand, alcohol mice nose-poked more in the water corner during the 4% phase suggesting a preference for the water corner as far as consumption is concerned (Fig. 1E), even though they drank significant amounts of alcohol throughout the whole training (10.2 ± 0.99 g/kg/day) (Fig. 1F). During the W, the alcohol mice increased activity in the reward corner, while the alcohol-naive mice performed less NPs in the reward corner compared to the last day of FA to alcohol (Fig. 1D). This indicates increased alcohol seeking in the alcohol group and extinction of seeking in the non-active corner by the alcohol-naive animals. During the CR, both the alcohol-naive and alcohol mice made more NPs in the reward corner, as compared to the last day of E before the test (Fig. 1E, left), indicating that the cue light attracted attention and was rewarding for the animals in both experimental groups. However, the alcohol mice showed a greater increase in the number of NPs during CR compared to the alcohol-naive group (Fig. 1E, right). Thus the light cues bore rewarding properties to both the alcohol-naive and alcohol mice, however, during periods of reward unavailability the alcohol mice showed higher interest in the reward-associated context and cues than the alcohol-naive animals.

### The analysis of AMPAR and NMDAR subunits in dDG of alcohol and alcohol-naive mice

**Fig. 2:**
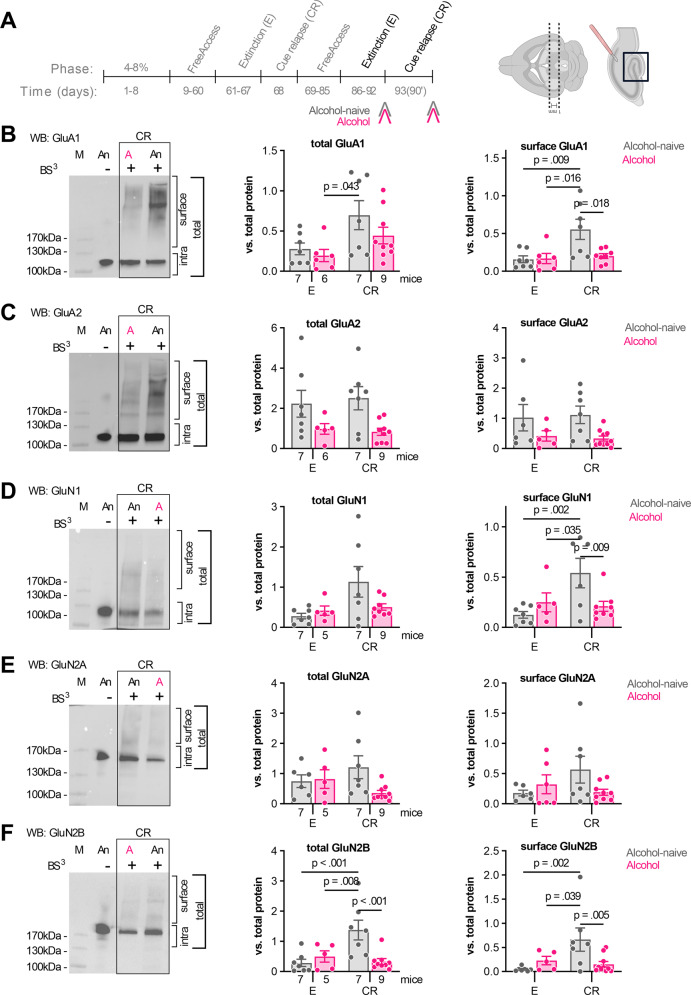
Upregulation of dDG AMPAR and NMDAR subunits during cue relapse is impaired in alcohol mice. **A** Experimental timeline. Alcohol mice (A, *n* = 14) and alcohol-naive animals (An, *n* = 15) were trained in the IntelliCages and sacrificed during E (day 92) or CR (day 93). Brain slices were used for the biochemical analysis of NMDAR and AMPAR subunits, analysis of dendritic spines and whole-cell patch recording of the granule cells in the dDG. For the biochemical analysis, DG was cut from a 1-mm thick slice of the dorsal hippocampus, chopped and incubated with BS^3^. Exemplary blots show the expression of AMPAR and NMDAR subunits. Samples were treated with BS^3^ (BS^3^+). BS^3^− samples are shown to present the band for non-modified protein. M, molecular weight marker. **B** GluA1 protein. (*left*) Representative blot and summary of data showing (*middle*) total GluA1 (two-way ANOVA, training: *F* (1, 25) = 7.730, *p* = 0.010; alcohol: *F* (1, 25) = 1.95, *p* = 0.174; training × alcohol: *F* (1, 25) = 0.508, *p* = 0.482) and (*right*) surface GluA1 (two-way ANOVA, training × alcohol: *F* (1, 24) = 5.34, *p* = 0.029). **C** GluA2 protein. (*left*) Representative blot. Summary of data showing (*middle*) total GluA2 (two-way ANOVA, training: *F* (1, 24) = 0.0174, *p* = 0.895; alcohol: *F* (1, 24) = 9.45, *p* = 0.005; training × alcohol: *F* (1, 24) = 0.194, *p* = 0.663) and (*right*) surface GluA2 (two-way ANOVA, training: *F* (1, 24) = 0.0002, *p* = 0.987, alcohol: *F* (1, 24) = 7.158, *p* = 0.013, training × alcohol: *F* (1, 24) = 0.117, *p* = 0.734). **D** GluN1 protein. (*left*) Representative blot and summary of data showing (*middle*) total GluN1 (two-way ANOVA, training: *F* (1, 23) = 4.893, *p* = 0.037; alcohol: *F* (1, 23) = 1.331, *p* = 0.260; training × alcohol: *F* (1, 23) = 3.17, *p* = 0.088)) and (*right*) surface GluN1 (two-way ANOVA, training × alcohol: *F* (1, 22) = 7.163, *p* = 0.013). **E** GluN2A protein. (*left*) Representative blot and summary of data showing (*middle*) total GluN2A (two-way ANOVA, training: *F* (1, 23) = 3.265e−005, *p* = 0.995; alcohol: *F* (1, 23) = 2.31, *p* = 0.142; training × alcohol: *F* (1, 23) = 3.20, *p* = 0.086) and (*right*) surface GluN2A (two-way ANOVA, training: *F* (1, 24) = 0.897, *p* = 0.353, alcohol: *F* (1, 24) = 0.704, *p* = 0.409; training × alcohol: *F* (1, 24) = 3.62, *p* = 0.068). **F** GluN2B protein. (*left*) Representative blot and summary of data showing (*middle*) total GluN2B (two-way ANOVA, training × alcohol: *F* (1, 24) = 9.79, *p* = 0.004) and (*right*) surface GluN2B (two-way ANOVA, training × alcohol: *F* (1, 25) = 7.089, *p* = 0.013). *p < 0.05; **p < 0.01; ***p < 0.001 by Tukey’s post hoc tests. Numbers of animals in the experimental groups are shown on the graphs (each dot represents one animal).

To check the effects of CR on dDG synaptic function we analyzed the expression of AMPA and NMDA receptor subunits. After the first CR (Fig. 1A, day 71), the animals continued to drink alcohol (days 72–86) and were sacrificed at two time-points: after alcohol extinction (E, day 92) or after the second 90-min cue relapse (CR, day 93) (Fig. 2A) (Supplementary Fig. S2), as we observed a generation of silent synapses in the dDG of the alcohol mice at this time point [11]. The dDG was carved from the dorsal hippocampus sections (Fig. 2A). The tissue was chopped and incubated with membrane-impermeant protein cross-linking reagent, bis(sulfosuccinimidyl)suberate (BS^3^), to crosslink extracellular protein domains [31]. The expression of the cell surface and total proteins (cell surface plus intracellular) was assessed on the blots and normalized to total protein loaded on TGX stain-free gels [29]. The expression of GluA1 and GluA2 subunits of AMPAR, and GluN1, GluN2A, and GluN2B subunits of NMDAR were analyzed (Fig. 2).

In the alcohol-naive CR group, the total and cell surface GluA1, cell surface GluN1, and total and cell surface GluN2B levels, were all increased, as compared to the alcohol-naive E animals (Fig. 2B, D, F). Moreover, the cell surface levels of GluA1, GluN1, and GluN2B were higher in the alcohol-naive CR mice, as compared to the alcohol CR group. No significant differences were found between the experimental groups in the total and surface GluA2, total GluN1, and total and surface GluN2A levels (Fig. 2C–E).

Overall, our data show that during CR, as compared to W, both total and surface levels of GluA1, GluN1, and GluN2B are significantly increased in the dDG of the alcohol-naive mice. These changes were not observed in the alcohol animals. These observations suggest that the synapses of the alcohol-naive, but not alcohol animals, were strengthened during CR. To test this hypothesis, we analyzed the morphology of dendritic spines (as a proxy of glutamatergic synapses) and the ratio of AMPAR and NMDAR EPSCs (as a measure of synaptic strength) in the granule cells of both alcohol-naive and alcohol mice.

### Morphological changes of dDG dendritic spines during cue relapse

**Fig. 3:**
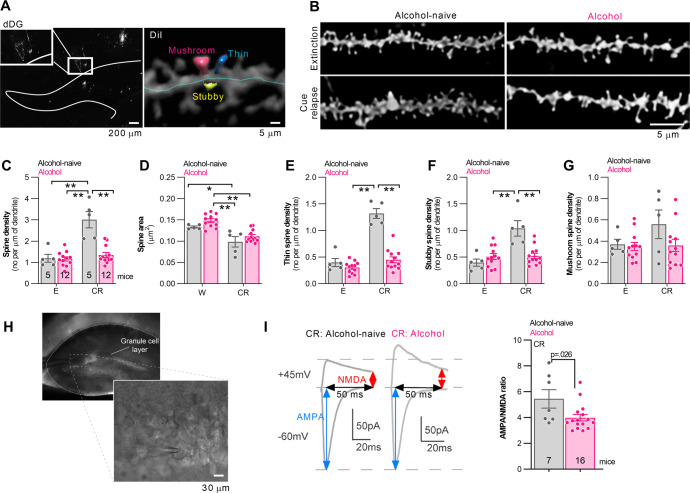
Cue relapse-induced generation of new dendritic spines in dDG is impaired in alcohol mice. **A** Diolistic labeling of dDG neurons. Alcohol-naive (An) and alcohol-trained (A) mice were trained in IntelliCages and sacrificed during extinction (W, day 92) and cue relapse (CR, day 93). Brains were sliced, and one half (random) of each slice was stained with DiI to visualize the morphology of dendritic spines in the dDG. Spines were categorized into 3 types: thin, stubby and mushroom. The second halves of brain slices were used for the patch-clamp electrophysiological analysis. **B–G** The summary of the morphologic analysis of dendritic spines. **B** Representative microphotographs of dendritic spines in the middle molecular layer of the dDG. **C** Density of dendritic spines (two-way ANOVA, alcohol × training: *F* (1, 30) = 20.79, *p* < 0.0001; Tukey’s post hoc tests: An CR vs. all groups, *p* < 0.01). **D** Dendritic spines areas (training: *F* (1, 29) = 32.56, *p* < 0.0001; alcohol: *F* (1, 29) = 4.893, *p* = 0.035; Tukey’s post hoc tests: A E vs. A CR and An CR, *p* < 0.01; An E vs. An CR, *p* = 0.012). **E** Density of thin spines (two-way ANOVA, alcohol × training: *F* (1, 30) = 38.61, *p* < 0.0001; Tukey’s post hoc tests: An CR vs. all groups, *p* < 0.01). **F** Density of the stubby spines (two-way ANOVA, alcohol × training: *F* (1, 30) = 16.92, *p* = 0.0003; Tukey’s post hoc tests: An CR vs. all groups, *p* < 0.01). **G** Density of mushroom spines (training: *F* (1, 30) = 2.002, *p* = 0.1674; alcohol: *F* (1, 30) = 2.45, *p* = 0.127). **H**, **I** The electrophysiological analysis of the granule cells. **H** The recording electrode was placed in the dDG and the stimulating electrode in the perforant path. **I** A representative averaged EPSCs elicited by stimulations at +45 mV (top) and −60 mV (bottom) is shown and AMPA/NMDA ratio was calculated 50 ms after pick response for the alcohol-naive and alcohol mice sacrificed during CR (Mann–Whitney *U* = 20, *p* = 0.026). *p < 0.05; **p < 0.01 by Tukey’s post hoc tests (**C**–**G**) or Mann–Whitney test (**I**). Numbers of animals in the experimental groups are shown on the graphs (each dot represents one animal).

Mice were trained in the IntelliCages and sacrificed after extinction (W, day 92) or cue relapse (CR, day 93) (Fig. 2A). We imaged dendritic spines of the dDG granule cells using diolistic staining of the brain sections [26], focusing on the dendrites in the medial part of the molecular layer (upper blade) that are innervated by the perforant path. Spine density and area, as well as the frequency of spines in three categories (thin, stubby and mushroom) that are thought to reflect spine maturity [32], were automatically assessed using NeuronStudio (Fig. 3A, B).

The density of dendritic spines was significantly upregulated in the alcohol-naive CR mice compared to the alcohol-naive E animals and alcohol CR groups (Fig. 3C). Moreover, the size of dendritic spines in the alcohol-naive CR group was smaller than in the alcohol-naive E group and alcohol E mice. The alcohol CR group also had smaller dendritic spines than the alcohol E group (Fig. 3D). These changes were associated with an increased density of thin and stubby spines in the alcohol-naive CR group, as compared to the alcohol E and CR mice (Fig. 3E, F). There was no significant effect of alcohol and time point on the density of mushroom spines (Fig. 3G). Thus, the dendritic spines analysis supports the hypothesis that CR results in increased connectivity of dDG neurons in the alcohol-naive mice resulting from a higher number of synapses located on thin and stubby dendritic spines, suggesting synaptic plasticity impairment in the alcohol mice.

### Functional changes of dDG circuitry during cue relapse

To test whether there are differences in synaptic function during CR between alcohol-naive and alcohol mice, we measured the ratio of AMPAR and NMDAR EPSCs. To this end, we performed whole-cell voltage clamp recordings from the granule cells of the upper blade of dDG while stimulating the perforant path (Fig. 3H). We observed a lower AMPA/NMDA ratio in the alcohol mice sacrificed during CR as compared to the alcohol-naive animals sacrificed at the same time point (Fig. 3I). Thus, the electrophysiological analysis of dDG granule cells matches our observation that the alcohol CR group GluA1 and GluN1 surface levels are decreased (more pronounced in GluA1 levels) compared to the alcohol-naive CR mice. It also indicates that the thin and stubby dendritic spines generated during CR in the alcohol-naive mice were likely mature. Thus, overall our data suggest that not only spinogenesis but also spine maturation are important components of CR-induced plasticity in alcohol-naive mice.

### The effect of ACA treatment on alcohol consumption and seeking

**Fig. 4:**
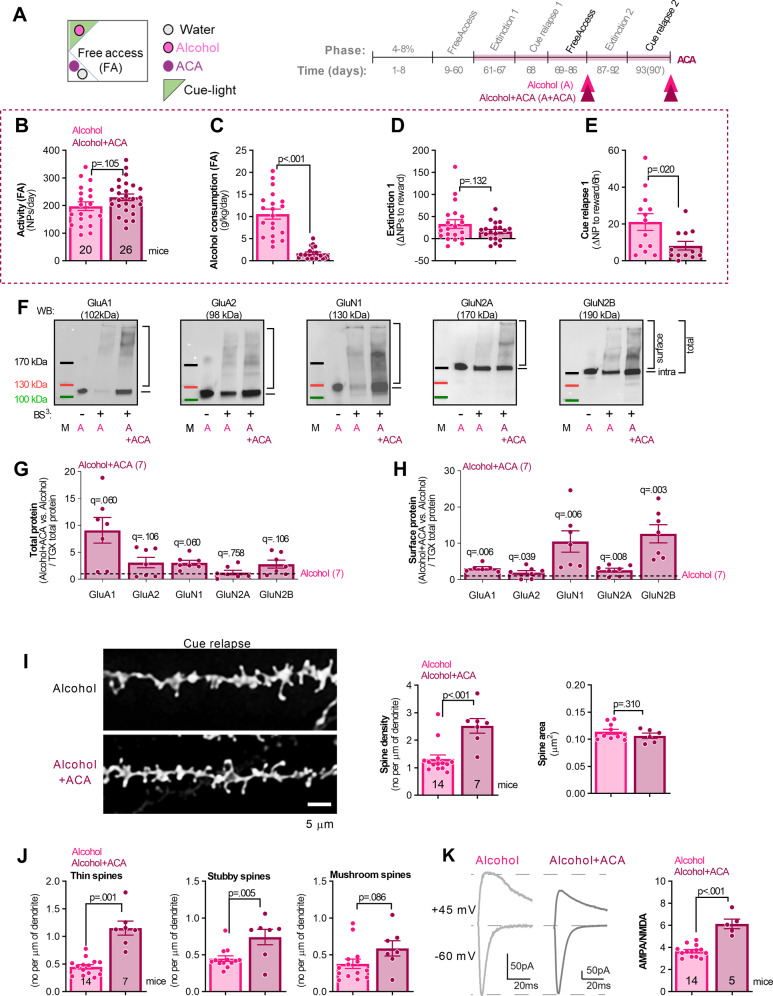
ACA decreases alcohol seeking during cue relapse and increases the density of dendritic spines and synaptic strength in dDG of alcohol mice. **A** Cage setup during ACA treatment and experimental timeline. Mice were trained to drink alcohol for 57 days (*n* = 35), and next treated with ACA (250 mg/kg/day) (day 58–93, *n* = 14) or non-treated (*n* = 21). They underwent alcohol E and CR during ACA treatment and were sacrificed during FA to alcohol or CR. Next their brains were sliced and used for protein analysis, patch-clamp electrophysiology recordings or stained with DiI to analyze dendritic spine morphology. **B–E** Behavioral effects of ACA treatment. **B** ACA had no effect on mice general activity (t(19) = 0.732, *p* = 0.472) and (**D**) alcohol seeking during E (t(19) = 1.164, *p* = 0.130), but (**C**) it decreased alcohol consumption during alcohol FA periods (t(19) = 4.95, *p* < 0.001), and (**E**) alcohol seeking during CR (t(19) = 2.55, *p* = 0.019). **F–H** Analysis of dDG AMPAR and NMDAR subunits after ACA treatment. **F** Exemplary western blots showing AMPAR and NMDAR subunits. Alcohol (A) samples without BS^3^ (BS^3^−) and samples with BS^3^ are shown. M molecular weight marker. **G** Summary of data showing total proteins (Two-way RM ANOVA with two-stage linear step-up procedure of Benjamini, Krieger and Yekutieli for multiple comparisons, main effect of ACA: *F* (1, 56) = 20.88, *p* < 0.001) and (**H**) surface expression of AMPAR and NMDAR subunits (main effect of ACA: *F* (1, 54) = 32.94, *p* < 0.001). Q values for the two-stage linear step-up procedure of Benjamini, Krieger, and Yekutieli for multiple comparisons are shown on the graphs. **I**, **J** The analysis of dDG dendritic spines during CR after ACA treatment. **I** Representative microphotographs and summary of data showing the effect of ACA on density (t(19) = 4.25, *p* < 0.001) and area of dendritic spines (t(19) = 1.54, *p* = 0.309). **J** ACA increased density of thin (t(19) = 6.65, *p* = 0.001), and stubby spines (t(19) = 3.19, *p* = 0.005), and had no effect on density of mushroom spines (t(19) = 1.81, *p* = 0.086). **K** Electrophysiological analysis of the dDG granule cells during CR after ACA treatment. Representative averaged EPSCs elicited by stimulations at +45 mV (top) and −60 mV (bottom) are shown. AMPA/NMDA ratio was calculated for the alcohol-trained and ACA-treated and non-treated mice sacrificed during CR (t(19) = 7.22, *p* < 0.001). *p < 0.05; **p < 0.01; ***p < 0.001 by *t* tests. Numbers of animals in the experimental groups are shown on the graphs (each dot represents one animal).

Since the alcohol mice sacrificed during CR have reduced cell surface expression of AMPAR and NMDAR, dendritic spine density and AMPA/NMDA ratio in the dDG compared to the alcohol-naive CR mice, we hypothesized that a pharmacological manipulation to decrease CR will enhance dDG function. Acamprosate (ACA) successfully dampens a hyper-glutamatergic state in the alcohol-dependent brain [33] and therefore normalizes alcohol consumption in mutant mice (with elevated extracellular glutamate levels, that drink more than wild-type littermates) and human alcoholics [24, 33]. Moreover, ACA prolongs periods of abstinence in human patients [34] and decreases alcohol seeking during CR in animal models [11, 28]. Accordingly, we hypothesized that ACA therapy would strengthen the compromised dDG circuit in the alcohol mice. The animals were trained in IntelliCages to drink alcohol for 57 days and were next randomly assigned to two experimental groups so they did not differ in the average activity and alcohol consumption before ACA treatment (Supplementary Fig. S3). Next, while still having access to alcohol, one group of mice drank ACA in the water corner (*n* = 26) (250 mg/kg/day), and the rest continued alcohol consumption without any treatment (*n* = 20) (d58-93). The animals were sacrificed either during FA to alcohol + ACA or after CR (Fig. 4A). ACA did not affect mice activity (Fig. 4B), but it decreased alcohol consumption (Fig. 4C), as well as alcohol seeking during CR (Fig. 4E), as compared to the non-treated mice. ACA did not significantly affect alcohol seeking during E (Fig. 4D, and Supplementary Fig. S4).

### The effect of ACA on dDG AMPAR and NMDAR levels in mice drinking alcohol

The analysis of AMPAR and NMDAR subunits expression showed that the ACA treatment on the alcohol mice significantly increased cell surface levels of all analyzed AMPAR and NMDAR subunits (Fig. 4H). There was also a significant effect of ACA on the total levels of the analyzed proteins, however, post hoc analysis did not show significant differences between Alcohol and Alcohol+ACA groups (Fig. 4G).

### The effect of ACA treatment on dendritic spines morphology and dDG synaptic transmission in alcohol mice

The analysis of dendritic spines revealed that the alcohol mice treated with ACA and sacrificed during CR had higher dendritic spine density as compared to the untreated alcohol animals. ACA treatment did not affect the mean size of dendritic spines (Fig. 4I). However, ACA treatment increased the density of thin and stubby spines, and had no significant effect on mushroom spines (Fig. 4J).

Moreover, the AMPA/NMDA ratio analyzed in dDG granule cells in the alcohol mice treated with ACA and sacrificed during CR was significantly increased compared to the non-treated alcohol mice (Fig. 4K). Thus, our data show that ACA treatment increased the expression of total and surface AMPARs and NMDARs, increased the density of thin and stubby dendritic spines and increased dDG synaptic transmission. These observations support the hypothesis that a strengthened dDG circuit by ACA treatment enables control and decreases impulsiveness during alcohol CR.

### The effect of ACA on alcohol-naive mice

To test the effect of ACA on the alcohol-naive mice, a new group of mice was trained in the IntelliCages. They never had access to alcohol (*n* = 15), and after 57 days of the training the animals were treated with ACA (*n* = 8) according to the same schedule as the alcohol mice (Fig. 4A). The remaining animals continued to drink only water (*n* = 7). ACA affected neither general nor activity in the cued corner during cue light E and CR (Supplementary Fig. S5). The animals were sacrificed during the FA period to analyze the expression of AMPAR and NMDAR subunits, and during CR to analyze the morphology and function of dDG synapses. ACA treatment did not affect the expression of total and cell surface AMPAR and NMDAR subunits (Supplementary Fig. S5E–G). We found no effect of ACA on the density and size of dendritic spines, as well as the density of thin, stubby and mushroom spines (Supplementary Fig. S5H, I). There was also no effect of ACA on the AMPA/NMDA ratio (Supplementary Fig. S5J). Thus, ACA treatment in alcohol-naive mice does not affect behavior, AMPAR and NMDAR levels, dendritic spines morphology and dDG synapse function.

### The effect of PSD-95 downregulation in dDG on alcohol-related behaviors

**Fig. 5:**
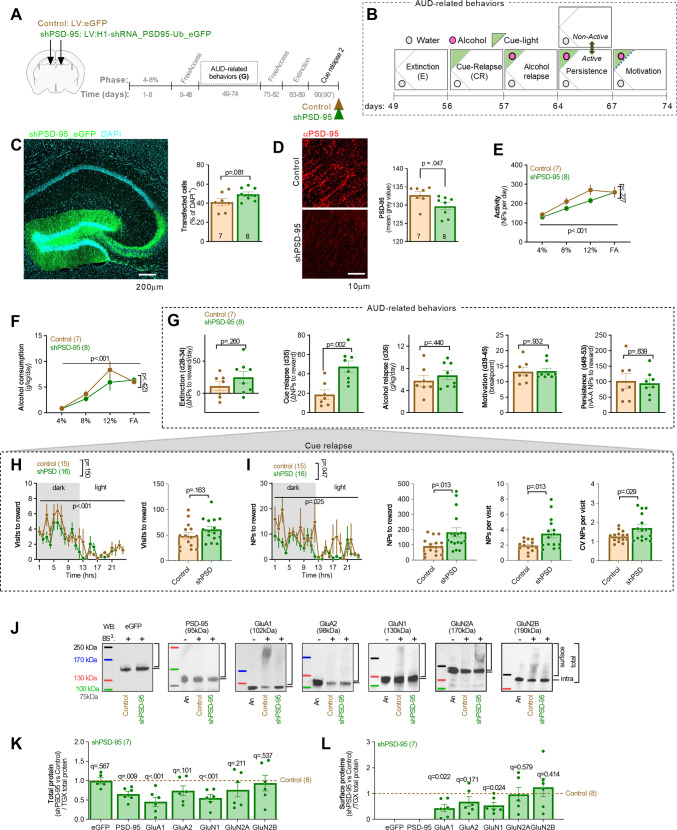
Silencing dDG PSD-95 expression with shRNA results in decreased expression of AMPAR and NMDAR subunits. **A**, **B** Experimental timeline and cage setups during alcohol-related tests. **A** shPSD-95 (*n* = 8) and Control (*n* = 7) LVs were injected into dDG. Two weeks later mice underwent training in the IntelliCages, (**B**) and alcohol-related behaviors were analyzed: alcohol seeking during extinction (E) and relapse induced by alcohol-predicting cues (CR), alcohol drinking during relapse, motivation to drink alcohol, and persistence in alcohol seeking. Mice were sacrificed after the 90-min CR (day 90). **C**, **D** Downregulation of PSD-95 levels by shRNA in dDG. **C** Exemplary picture showing viral infection in dDG (left) and summary of data showing no difference in fraction of infected cells (t(13) = 1.09, *p* = 0.081; section/mouse, Control = 14/7; shPSD-95 = 16/8). **D** Exemplary picture and summary of data showing significant downregulation of PSD-95 levels by shPSD-95, as compared to the Control virus (t(13) = 2.95, *p* = 0.047; sections/mice, Control = 51/7; shPSD-95 = 42/8). *p < 0.05. **E**, **F** Summary of data showing the effect of dDG shPSD-95 on (**E**) general mice activity (RM ANOVA, *F* (1, 13) = 1.61, *p* = 0.23) and (**F**) alcohol consumption (*F* (1, 13) = 0.68, *p* = 0.42). **G** Summary of data showing the effect of dDG shPSD-95 on alcohol-related behaviors: alcohol seeking during E (*t* test: t(13) = 1.18, *p* = 0.26); alcohol seeking during CR (t(13) = 3.81, *p* = 0.002); alcohol consumption during alcohol relapse (t(13) = 0.797, *p* = 0.44); motivation to drink alcohol (t(13) = 0.087; *p* = 0.93). and persistence in alcohol seeking (t(13) = 0.21, *p* = 0.84). **p < 0.01. **H** Summary of data showing visits to the alcohol corner during CR in 1-h bins (RM ANOVA, effect of time: *F* (6.915, 196.0) = 9.17, *p* < 0.001; effect of shPSD-95: *F* (1, 29) = 2.19, *p* = 0.149) and during the whole test (t(28) = 1.433, *p* = 0.163). **I** Summary of data showing NPs to the alcohol corner during CR in (**J**) 1-h bins (RM ANOVA, effect of time: *F* (5.473, 155.1) = 2.56, *p* = 0.025; effect of shPSD-95: *F* (1, 29) = 4.29, *p* = 0.047), during the whole test (Welch’s t test, t(22.55) = 2.709, *p* = 0.013), NPs per visit (Welch’s t test, t(17.51) = 2.779, *p* = 0.013) and coefficient of variation (CV) of NPs per visit (Welch’s t test, t(18.53) = 2.356, *p* = 0.029). **J–L** Analysis of AMPAR and NMDAR subunits in dDG of the Control (*n* = 7) and shPSD-95 mice (*n* = 7) during CR. **J** Exemplary western blots showing AMPAR and NMDAR subunits. Alcohol-naive (An) samples without BS^3^ (BS^3^−) and samples with BS^3^ (Control and shPSD-95) are shown. **K**, **L** Summary of data showing total proteins (Two-way RM ANOVA with two-stage linear step-up procedure of Benjamini, Krieger, and Yekutieli for multiple comparison, main effect of shPSD-95: *F* (1, 14) = 10.70, *p* = 0.0056) and surface proteins (shPSD-95 × protein: *F* (4, 46) = 9.986, *p* < 0.001).

We hypothesized that the molecular manipulations that impair dDG synaptic transmission will potentiate CR. To test this hypothesis we used dDG-targeted lentiviral vectors (LVs) carrying short hairpin RNA for *DLG-4* mRNA (coding PSD-95 protein) under human H1 promoter cassette (shPSD-95) with an enhanced green fluorescent protein (eGFP) for detection (Fig. 5A) [27]. We chose this viral vector delivery strategy as in vitro hippocampal neurons studies showed that downregulation of PSD-95 by shRNA decreases AMPAR EPSCs and the ratio of AMPA/NMDA EPSCs [27, 35–37], increases the frequency of silent synapses (lacking functional AMPAR) [38] and arrests spine morphological development (decreased density and maturation) [36]. Importantly, we observed that alcohol E downregulates PSD-95 levels in the dDG (Supplementary Fig. S5) and our previous work showed that CR results in the generation of silent synapses in the dDG of alcohol mice, and this phenomenon is more pronounced in mice with an AUD-like phenotype [11].

shPSD-95-GFP, or a Control LV coding eGFP only, were bilaterally delivered to the dDG 2 weeks before alcohol training (Fig. 5A). At the start of the longitudinal study, mice had unlimited access to alcohol in increasing concentrations (4 and 8%, and 10% during FA; days 1–27), followed by the assessment of alcohol seeking during extinction (W, days 28–34) and CR (day 35). Three other behaviors that resemble DSM-V criteria for AUD [1] were also measured based on our previously published protocol [25]: loss of control over alcohol consumption measured as alcohol consumption (g/kg/day) during alcohol relapse (Alcohol relapse, day 36), high motivation to drink alcohol measured in a progressive-ratio schedule of reinforcement as reaching a breakpoint (Motivation, days 39–45) [39], and persistence of alcohol seeking even during signaled alcohol non-availability [40] measured as the change in the number of NPs to the alcohol corner during the non-active vs. active phases of the test (Persistence, days 49–53) (Fig. 5B).

Post-training analysis confirmed that both the shPSD-95 and Control virus transduced ~40% of the dDG cells and shPSD-95 significantly decreased PSD-95 expression in the dDG (Fig. 5C, D). shPSD-95 did not affect the general activity and alcohol consumption of mice (Fig. 5E, F). shPSD-95 also did not affect alcohol seeking during W, alcohol consumption during alcohol relapse, motivation to drink alcohol, and persistence in alcohol seeking (Fig. 5G). However, silencing of PSD-95 expression significantly increased alcohol seeking induced by the presentation of alcohol-predicting cues (Fig. 5G). Thus, the dDG-targeted manipulation was specific to CR and did not affect other AUD-related behaviors.

### The effect of PSD-95 downregulation in dDG on cue relapse

Since shPSD-95 mice, as compared with the controls, performed more NPs to the reward corner during CR despite no difference between the experimental groups in motivation or craving for alcohol, we hypothesized that dDG shPSD-95 impairs the updating of predictive reward cue value, extinction of reward seeking and/or increases impulsivity, which are traits that may potentially affect CR [6, 41, 42]. To test these hypotheses we focused on mice behavior during CR. We observed that dDG shPSD-95 did not affect the number of visits to the reward corner during the test (Fig. 5H), indicating that the manipulation did not impair the decision to enter the corner. Mice in both groups performed more visits in the reward corner at the beginning of the test and decreased the frequency of visits during the test (Fig. 5H). Thus, the recall and extinction of reward cue memory were not significantly altered by dDG shPSD-95. However, shPSD-95 mice not only made more NPs to the reward corner, as compared to the control group, but they also made more NPs per visit and showed higher variation in the number of NPs per visit as compared to the control animals (Fig. 5I). Altogether, shPSD-95 mice failed to terminate non-reinforced reward seeking and their behavior was more erratic.

### The effect of PSD-95 downregulation on AMPAR and NMDAR levels

To test the effect of shPSD-95 on NMDAR and AMPAR levels in the dDG, a second cohort of mice underwent surgery and alcohol training. After the AUD-related tests, they continued to drink alcohol, underwent a second extinction (W2) and were sacrificed after the second 90 min cue relapse 2 (Fig. 5A, CR2, day 90). dDGs were excised and prepared for the biochemical analysis of NMDAR and AMPAR levels with BS^3^. Similar to the first cohort, we observed a specific impairment of alcohol seeking during CR2, and no effect of shPSD-95 on other AUD-related behaviors (Supplementary Figs. S6, S7). Analysis of eGFP expression confirms similar levels of the shPSD-95 and control virus transduction (Fig. 5J, K). shPSD-95 significantly decreased total levels of PSD-95, GluA1 and GluN1, and cell surface levels of GluA1 and GluN1 (Fig. 5L). Thus, the long-term reduction of dDG PSD-95 levels downregulated both the expression and cell surface anchoring of GluA1 and GluN1 subunits during CR2.

## Discussion

The neuronal mechanisms that control an individual’s response to alcohol-predicting environmental cues are not fully understood. It is also unknown how they differ from the mechanisms that drive responses to neutral cues. Here, we observed that *the* presentation of cue light to the alcohol-naive mice resulted in increased animal interest in the cued cage corner and strengthening of the dDG circuit. Surprisingly, CR-induced synaptic plasticity was impaired in the alcohol mice, as compared to the alcohol-naive animals. Moreover, ACA treatment of the alcohol mice rescued impaired dDG connectivity and reduced alcohol consumption and seeking; while decreased synaptic transmission by dDG-targeted shPSD-95 enhanced impulsive alcohol seeking during CR, but did not affect alcohol reward, motivation for alcohol or anxiety. Overall, we showed that reinstatement of alcohol seeking induced by alcohol-predicting cues is controlled by dDG synaptic transmission as such that impaired transmission predicts enhanced reinstatement of alcohol seeking.

### The role of synaptic plasticity in dDG in cue relapse

dDG synaptic plasticity is believed to underlie hippocampus-dependent memory formation, recall, extinction and updating [43–48]. In particular, visual cues that drive animal behavior and learning, potentiate the perforant pathway from the medial entorhinal cortex, layer II, to the DG [49]. Presentation of the cue light during CR in alcohol-naive mice resulted in increased expression of total and cell surface GluA1, GluN1 and GluN2B proteins and spinogenesis in dDG, as compared to the cue extinction. In particular, CR increased the frequency of thin and stubby dendritic spines with large heads suggesting that synaptogenesis and dendritic spine maturation are important components of CR-induced plasticity in alcohol-naive mice. This conclusion is also supported by an increase in synaptic strength (higher AMPA/NMDA ratio).

The exact function of CR-induced synaptic plasticity remains unknown. We think that it is unlikely to support cue memory formation or recall as reward cue memory was formed and recalled even by the animals with impaired dDG plasticity, as substantiated by CR observed in the alcohol mice with dDG shPSD-95 expression. Alternatively, synaptic plasticity during CR may be required to update the value of the predictive reward cue when the reward corner was inactive. This hypothesis is supported by findings that DG inactivation impaired the ability to learn changed contingencies in a previously encountered environment [43, 47, 48], as well as our observations that impairment of dDG synaptic transmission by shPSD-95 potentiates non-reinforced alcohol seeking during CR. In particular, shPSD-95 mice performed more NPs in the reward corner per visit and the number of NPs per visit was more variable. Thus, these animals failed to update the value of the predictive cue within a visit and behaved more impulsively than the alcohol-naive control mice. On the other hand, the shPSD-95 animals did not differ from the controls in the number of visits to the reward corner, and the frequency of visits in both groups decreased throughout the test duration. Hence they extinguished the reward cue memory between the visits. The role of aberrant synaptic plasticity of dDG in the regulation of alcohol CR is in agreement with our earlier study showing that CR results in the generation of silent synapses in the dDG, and this process is enhanced in the mice with addiction-like phenotype (with intensive CR), as compared to mice with an addiction-resistant phenotype [11]. Importantly, shPSD-95 increases the frequency of silent synapses [45]. Thus, overall our data suggest that impaired PSD-95-dependent synaptic transmission during CR, including generation of silent synapses, results in an addiction-like phenotype, characterized by enhanced and impulsive alcohol seeking. Interestingly, fMRI studies found that the functional connectivity of the hippocampus in the resting-state is reduced in alcoholics and drug addicts, as compared to healthy individuals [50, 51].

On the other hand, our observations seem to contradict earlier studies showing increased Fos expression and synaptic transmission in the DG [52, 53], as well as enhanced synaptic plasticity in the NAc upon relapse induced by drug-associated cues [5, 13, 14]. We propose that this discrepancy may stem from several differences in the experimental conditions used in these former publications and our study. Firstly, in the earlier studies the authors analyzed the molecular and synaptic processes occuring in the drug-trained animals that were exposed to the drug-associated stimuli, but did not include a drug-naive group exposed to neutral sensory cues [52, 53]. Thus, these authors were not able to compare the differences in synaptic processes evoked by neutral and drug-associated discrete cues. Secondly, in the former studies CR was combined with an exposure to a novel context [52], which on its own activates Fos expression in the DG [54]. Hence, Fos expression could be induced by a combination of contextual and cue information. Here, mice were housed in the IntelliCages and had constant access to the inactive alcohol corner during W. During CR, only alcohol-predicting cues were re-activated. Thirdly, in the previous studies, the authors used drug-associated cues (cues consistently paired with drug reward) [5, 13, 14], while in our experiments the light cue predicted the availability of alcohol and therefore served more as a discriminative stimulus (a stimulus that informs an organism when and where a particular action will result in a specific reward). Although the differences in the neuronal networks involved in the processing of drug-associated and drug-predicting cues are poorly understood, they likely exist [7] and should be carefully studied in future experiments. This point is important as abstinent alcoholics are sensitized to alcohol-predicting cues, and these cues may become a significant threat to abstinence. Furthermore, the synaptic changes induced by drug-associated cues in the NAc are very rapid and transient as synaptic growth is observed within 15 min of the CR, and is reversed 30 min later [5, 13, 14]. Here, we tested the dDG circuit 90 min after the CR (as this is the time point we observed the generation of silent synapses in the alcohol mice [10, 11]), so we cannot exclude the possibility that synaptic strengthening of the dDG circuit may occur during CR in the IntelliCages at an earlier time point than when we tested. Finally, the key differences likely exist in how drug cues are processed by the NAc and dDG synapses.

### The mechanisms of impaired dDG plasticity after alcohol

The cellular mechanisms that result in the generation of silent synapses [11] and dDG plasticity impairment in alcohol mice are unknown. However, the existing literature proposes several possibilities that are worth considering. Firstly, both drugs of abuse and alcohol compromise DG neurogenesis [15–17]. As a likely consequence of impaired neurogenesis, the overall number of granule cells was shown to be decreased both in rats drinking alcohol [19] and in human patients diagnosed with AUD [20]. Thus, enhanced CR in the alcohol mice may result from impaired dDG circuit connectivity. Here, we analyzed the expression of AMPAR and NMDAR subunits, as well density and size of dendritic spines in the dDG and none of these parameters were changed in the alcohol mice during W, as compared to the alcohol-naive animals. Thus, reduced dDG neurogenesis and connectivity before CR are unlikely reasons for the impaired CR in our experimental model.

Secondly, both reward presentation and dopamine (DA) release inhibit the activity of DG granule cells [55, 56]. In particular, DA release and stimulation of D2 receptors in the DG induce long-term depression of cortical inputs, diminish theta oscillations, and suppress hippocampus-dependent memory formation [55, 56]. Thus, D2 receptor-dependent signaling may control the dDG circuit plasticity and generation of silent synapses during CR. Similarly, morphine induces silent synapses in D2-type NAc neurons via the internalization of AMPA receptors from pre-existing synapses [57]. Importantly, the inhibitory effects of DA are specific for DG, as DA enhances LTP at the CA3–CA1 synapses [58] and likely due to postsynaptic D2 receptors being exclusively expressed in DG neurons [59]. The role of DA in the regulation of dDG plasticity during CR in the alcohol mice remains to be validated in future experiments.

Thirdly, long-term alcohol use results in increased extracellular levels of glutamate as well as impairments of glutamate transport and signaling [60–62]. Thus, the impaired CR-induced synaptic plasticity in the alcohol mice could result from glutamatergic system dysregulation. This hypothesis is corroborated by the observation that ACA, a weak NMDAR antagonist or more potent mGluR antagonist [63, 64], attenuates CR [64]. It also upregulates the expression of AMPA and NMDA receptors during FA to alcohol, as well as increases the density of dendritic spines and AMPA/NMDA ratio in the alcohol mice during CR to levels observed in the alcohol-naive animals. The mechanisms that lead to the attenuation of CR by ACA are unknown. However, since both depleted dDG NMDAR levels and impaired synaptic transmission correlated with enhanced CR, we favor the mechanism that involves ACA antagonism on mGluR signaling to dampen CR. This hypothesis is also supported by the observation that the mGluR5 antagonist MPEP reduces ethanol-seeking and relapse [65].

Overall, our data indicate that the increased dDG synaptic transmission controls CR. Additionally, dDG glutamatergic synapses do not affect motivation to drink alcohol, alcohol craving during E or persistence in alcohol seeking. The dDG role in regulation of anxiety is also unlikely. The review of published data and ours support the hypothesis that the dDG updates the value of the predictive reward cue and regulates impulse. The CR-induced synaptic processes are impaired in the alcohol mice, resulting in the generation of silent synapses, as well as enhanced and impulsive alcohol cue responses. Thus drugs that restore synaptic plasticity in the individuals that abuse alcohol may help to control AUD-related behaviors. As our study included only female mice, future experiments to investigate whether similar synaptic processes are observed in males during alcohol seeking will be of interest.

## Supplementary information

SUPPLEMENTAL MATERIAL
