# Supplementary material for: Impaired synaptic transmission in dorsal dentate gyrus increases impulsive alcohol seeking
Source: Neuropsychopharmacology. 2022 Oct 1;48(3):436–47. doi: 10.1038/s41386-022-01464-5 (PMC9852589; doi:10.1038/s41386-022-01464-5)
Supplement: Supplementary file 1 — SUPPLEMENTAL MATERIAL [file 41386_2022_1464_MOESM1_ESM.docx]

Title: **Impaired synaptic transmission in dorsal dentate gyrus**

**increases impulsive alcohol seeking.**

**Running title**: Dentate gyrus controls cue relapse

**Authors**: Maria Nalberczak-Skóra PhD ^1,2^, Anna Beroun PhD ^3^, Edyta Skonieczna MSc ^1^, Anna Cały PhD ^1^, Magdalena Ziółkowska MSc ^1^, Roberto Pagano ^1^, Pegah Taheri MSc ^1^, Małgorzata Piechota PhD ^1^, Katarzyna Kalita PhD ^3^, Ahmad Salamian ^1^ and Kasia Radwanska PhD ^1^*

^1^ Laboratory of Molecular Basis of Behavior, Nencki Institute of Experimental Biology of Polish Academy of Sciences, 3 Pasteur St., Warsaw 02-093, Poland;

^2^ Experimental Psychopathology Lab, Institute of Psychology of Polish Academy of Sciences; 1 Jaracza St., Warsaw 00-378, Poland.

^3^ BRAINCITY, Nencki Institute of Experimental Biology of Polish Academy of Sciences, 3 Pasteur St., Warsaw 02-093, Poland.

***Corresponding author:** Kasia Radwanska, Ph.D., Laboratory of Molecular Basis of Behavior, the Nencki Institute of Experimental Biology of Polish Academy of Sciences, 3 Pasteur St., Warsaw 02-093, Poland; e-mail: k.radwanska@nencki.edu.pl; tel: +48501736942;

**Keywords**: AMPAR, NMDAR, dendritic spines, synaptic strength, AMPA/NMDA ratio, PSD-95, acamprosate, alcohol addiction, dentate gyrus, IntelliCages.

**SUPPLEMENTARY MATERIALS AND METHODS**

***Animal model of alcohol addiction in the IntelliCages.***

IntelliCages. After 1 week of acclimatization, the mice were injected (s.c.) with unique microtransponders (11.5 mm length, 2.2 mm diameter; Trovan, ID-100) under brief isoflurane anesthesia. The mice were then allowed to recover for 3 days, and the animals with properly located microtransponders were introduced to the IntelliCage system (NewBehavior AG, Zürich, Switzerland) (http://www.newbehavior.com/), 15 animals per system. The IntelliCage consists of a large standard rat cage (20.5 cm high, 40 cm x 58 cm at the top, 55 cm x 37.5 cm at the base). In each corner, a triangular learning chamber is located with two bottles. To drink, only one mouse can enter a plastic ring (outer ring: 50 mm diameter; inner ring: 30 mm diameter; 20 mm depth into outer ring) that ends with two 13 mm holes (one on the left, one on the right) that gives access to bottle nozzles. Each visit to the corner, NP at the doors governing access to the bottles, and licks were recorded by the system and ascribed to a particular animal.

The training in the IntelliCage was composed of the following phases: initiation of alcohol consumption in increasing concentrations (4-12%), free access to 12% alcohol (FA), progressive ratio test (Motivation), an alternating long access self-administration and extinction regimen (Persistence), extinction (E), cue relapse (CR) and alcohol relapse. “Alcohol-naive” and “Alcohol” mice were trained in separate cages.

*Adaptation phase.* All mice had free access to all bottles with water in both active corners. All doors were open. After 24 hours, when all mice visited and licked from both corners, the doors in the corners were closed. Under a fixed ratio of reinforcement (FR1), each nose-poke (NP) to the door opened the door, and gave a 5-second access to the bottles with water (nose-poke adaptation, NPA).

*Initiation of alcohol consumption and free alcohol access periods.* During the initiation of alcohol consumption (4-8%, days 1-8) and free alcohol access periods (FA), two corners were active, with two bottles each. In one corner, the animals had access to water (“water corner”), and in the other (“reward corner”) animals had access to an ethanol solution (Alcohol group) or water (Alcohol-naive mice). When alcohol (or water) was available in the “reward corner”, it was signaled by a green light turned on each time a mouse entered the corner. Each NP in the cage corners gave access to the bottles under FR1. During the 4-8% period animals had access to an ethanol solution in increasing concentrations (4 and 8% ethanol changed every 4 days, prepared from 96% ethanol and tap water). During the FA, 12% alcohol was available in the reward corner based on the maximal alcohol consumption in g/kg/day during the initiation of alcohol consumption. Daily alcohol consumption (g/kg/day) was calculated with the following formula: (number of licks of 12% alcohol per day x lick volume x 0.12 x 1 g/ml) / animal weight). To calculate the average lick volume, water consumption (in μl) was measured for 3 consecutive days. The average volume of one lick was measured as the total volume consumed / number of licks. According to these calculations, an average lick volume was established as 1.94 ± 0.2 μl.

*Motivation for alcohol.* During the progressive ratio test (Motivation), two corners were active and available to animals. The animals had to perform an increasing number of NPs (2, 4, 8, 12, 16, 20, 24, 28, 32, and 36) spaced by less than 1 s during one visit to open the door and be allowed for a 5 s access to the reward bottles. The number of required instrumental responses (NPs) increased when an animal performed 10 sets of responses of a given ratio. The tests were terminated when 90% of animals did not change FR (fixed ratio between number of NPs and reward access) level during the last 24 hours. The FR level reached during the test was used as an index of motivation.

*Persistence in reward seeking* was measured in an alternating long access self-administration and extinction regimen. Each test lasted 3 days, starting at the beginning of the dark phase and was composed of six, 6-hour long “active periods” (A) altered with 6-hour long “non-active periods” (nA). “Active” periods (A) were signaled by the green cue light in the reward corner. During the “active” periods, NPs at all doors opened the door for 5 s (FR 1). The “non-active” periods were signaled by elimination of the green cue light. During the “non-active” periods, NPs on the reward side were not followed by any scheduled consequences. Number of “reward” NPs performed during the test, as well as the difference of NPs performed during nA and A reward periods, were used as indices of persistence.

*Extinction of alcohol seeking followed by cue and alcohol relapse.* Extinction periods were signaled as the “no-reward” periods and lasted 7 days. The door to the reward was closed and NPs to the reward door were without scheduled consequences. Average daily number of NPs performed in the “reward corner” during the extinction, and a difference in the average number of NPs during the extinction vs. the last day before the test, were used as indices of alcohol seeking during extinction. Each extinction was followed by a 24-hour cue-relapse. A green cue light (reward-predicting cue) in the reward corner was presented each time a mouse entered the reward corner. However, NPs to the reward doors had no scheduled consequences. Average daily number of NPs performed in the “reward corner” during CR, and a difference in the average number of NPs during CR vs. the last day of extinction, were used as indices of alcohol seeking during CR. This test was followed by the reward relapse when bottles with reward (alcohol or water) were added into the active “reward corner”. During the test each NP into the reward door opened the door for 5 s. The amount of reward drank during the first day of relapse (number of licks) was used as an index of relapse.

Each test started (and finished) at the beginning of the dark phase (9:00 am). During extinction, the animals were sacrificed after 6 days of extinction plus 90 minutes (10:30-11:00 am). During CR2 the animals were sacrificed 90 minutes after the first visit in the reward corner (day 93).

***Tissue preparation for Western blot***

Mice were decapitated under isoflurane anesthesia, hippocampi were isolated and sliced into 1 mm-thick slices. dDG was cut from the slices with a razor blade. The dDG tissue was chopped and incubated with rotation for 30 minutes at +4 °C in artificial cerebrospinal fluid (aCSF) with a membrane-impermeant protein cross-linking reagent, bis(sulfosuccinimidyl)suberate (BS^3^) as previously described [[30]](https://www.zotero.org/google-docs/?ARIsxs) with modifications. Due to the large number of animals used in our study, we repeated the procedure several times and pooled the animals. To minimize the effect of tissue preparation, we sacrificed animals from at least two groups each round. This procedure allows for the distinction of the intra- and extracellular proteins, as extracellular, BS^3^-labeled proteins are heavier. The BS^3^ crosslinking was stopped by an addition of 1M glycine (BioShop, GLN001.1) and tissue was transferred into the ice-cold lysis buffer (25 mM HEPES, pH 7.4 (Acros Organics, ACRS17257); 500 mM NaCl (Gilbert, 6149214); 2mM EDTA (Invitrogen Life Technologies, 15575-038); 20 mM NaF (Merck, 201154); 1× protease inhibitor cocktail tablet (Roche, 5892791001); 0.1% (v/v) Nonidet P-40 (Sigma, 98379-6x). After sonication and centrifugation (20000× g at 4 °C) the supernatant was stored at -80 °C until further analysis.

***Western blot***

Equal amounts of total protein from each sample were mixed with a Laemmli buffer containing DTT (50 mM) and left to denature at 70°C for 10 minutes. The mixture was loaded on TGX precast gel wells, that contain trihalo compounds allowing stain-free visualization of total proteins (Bio-Rad #4568083), and ran until the loading buffer reached the bottom of the gel. The analyzed protein levels were normalized to the total protein levels. The membranes were blocked by 5% or 10% (depending on the antibody) milk diluted in TBST (Tris-buffered saline with Tween 20), and incubated with the primary antibody (GluA1 1:1000 Abcam #109450; GluA2 1:5000 Abcam #133477; GluN1 1:1000 Sigma #G8913; GluN2A 1:2000 Merck/Millipore #05-901R; GluN2B 1:3000 Abcam #183942; GluN3A 1:1000 Merck/Millipore #07-356) for 12 hours. After washing in TBST the membranes were incubated in a secondary antibody with HRP (1:5000, Vector pI-1000) and washed again. The membranes were visualized by G-Box apparatus using a chemiluminescent reagent (Advansta, K-12042-D10).

***Immunohistochemistry***

Mice were anesthetized and transcardially perfused with filtered PBS (Sigma-Aldrich) and 4% PFA (Sigma-Aldrich)/PBS. Brains were left overnight in the same fixing solution and transferred to 30% sucrose in PBS for 72 hours. Coronal brain sections (40 µm) of perfused mice (Leica CM1950 Cryostat, Leica Biosystems) were stored at -20 °C in anti-freeze buffer [PBS, 20% sucrose (Sigma–Aldrich), 15% ethylene glycol (Sigma–Aldrich), 0.05% NaN3 (Sigma–Aldrich)]. After washing from the buffer and incubation with 5% NDS (Jackson immuno research, 017-000-121) (in PBS), the slices were incubated with primary antibody (PSD-95, Millipore, #MAB1598, 1:500; GFP, Synaptic Systems #132004; 1:2000) in TBS with 0.3% Triton (TBST) and 5% NDS. The next day, the sections were washed with TBST and incubated with secondary antibodies (Invitrogen Alexa Fluor 488 #1182671 and 555 #1736967). Next, the slices were washed with PBS, mounted on microscopic slides and covered with DAPI containing medium (Southernbiotech, #00-4959-52). The fluorescent staining in the upper blade of dDG was photographed with a confocal microscope (Zeiss LSM800, magnification 63x).

***Electrophysiology***

Whole-cell patch-clamp technique was used to analyze AMPA/NMDA EPSCs ratio. Brains from decapitated mice were quickly submerged in the ice-cold cutting solution (135 mM NMDG, 1 mM KCl, 1.2 mM KH_2_PO_4_, 1.5 mM MgCl_2_, 0.5 mM CaCl_2_, 20 mM choline bicarbonate, 10 mM D-glucose, bubbled with carbogene – 5% CO2, 95% O2). Coronal 250 µm-thick slices were prepared using Leica VT1000 S vibratome. Slices containing DG were collected into a chamber filled with artificial cerebrospinal fluid (ACSF,119 mM NaCl, 2.5 mM KCl, 1 mM NaH_2_PO_4_, 26 mM NaHCO_3_, 1.3 mM MgCl_2_, 2.5 mM CaCl_2_, 10 mM D-glucose, bubbled with carbogen) and incubated for at least 30 min at room temperature. Slices were then transferred to the recording chamber, perfused with ACSF solution heated up to 31°C. A stimulating electrode was placed in the perforant path. Granule cells of the upper blade of dorsal DG were identified visually and patched with borosilicate glass capillaries (4–6 MΩ resistance) filled with internal solution (130 mM Cs gluconate, 20 mM HEPES, 3 mM TEA-Cl, 0.4 mM EGTA, 4 mM Na_2_ATP, 0.3 mM NaGTP, and 4 mM QX-314Cl, pH = 7.0–7.1, osmolarity: 290–295 mOsm). Series and input resistances were monitored throughout the experiment. Electrical stimulation was elicited by TTL pulse every 5 s. Recorded currents were filtered at 2 kHz (npi amplifiers) and digitized at 10 kHz (ITC-18 InstruTECH/HEKA). All recordings were performed in the presence of 50 μM picrotoxin (Abcam) in ACSF, to pharmacologically block inhibitory neurotransmission and focus on the excitatory pathway specifically. After reaching the whole-cell configuration, baseline currents were recorded for 2-3 minutes to ensure the stability of recorded amplitudes. AMPA receptor-mediated excitatory postsynaptic currents (EPSCs) were recorded at -60 mV and their amplitudes were measured at the peak of each current. NMDAR-mediated EPSCs were recorded at +45 mV and their amplitudes were measured at 50 ms after the peak, to ensure the absence of the AMPAR-mediated component. For both AMPARs and NMDARs EPSCs, 30-100 stable sweeps were recorded and averaged.

***Stereotaxic surgery***

Mice were anaesthetized with isoflurane (5% for induction, 1.5-2.0% after), fixed in the stereotaxic frame (51503, Stoelting, Wood Dale, IL, USA), and their body temperature was maintained using a heating pad. Stereotactic injections were performed bilaterally into the dDG region of the hippocampus using coordinates from the Bregma: ML, ±1.0 mm; AP, -2.0 mm; DV, -2.0 mm (Paxinos and Franklin, 2001). 0.5 µl of virus solution was microinjected through a beveled 26 gauge metal needle, attached to a 10 µl microsyringe (SGE010RNS, WPI, USA) connected to a microsyringe pump (UMP3, WPI, Sarasota, USA) and its controller (Micro4, WPI, Sarasota, USA), at a rate 0.1 µl/min. The needle was left in place for an additional 10 minutes following injection to prevent leakage of the vector. Mice were injected with lentiviral vectors (LVs) coding short-hairpin RNA silencing PSD-95 expression (H1-shRNA_PSD95-Ub-eGFP (0.5 µl/ site, viral titer 1.8 × 10^7^ gc/µl)) (gift from Dr. Oliver M. Schlüter, European Neuroscience Institute Göttingen, Germany) (Schlüter et al., 2006), and a control GFP-coding FUGW vector (Plasmid 14883, Addgene) (viral titer: 1.16 × 10^7^ gc/µl) were used. The viruses were prepared by the Laboratory of Animal Models at Nencki Institute of Experimental Biology, Polish Academy of Sciences. After the surgery, animals were given 14 days to recover before training in the IntelliCages. After training, the animals were perfused with 4% PFA in PBS and brain sections from the dorsal hippocampus were immunostained for PSD-95 and imaged with Zeiss Spinning Disc confocal microscope (magnification: 10x) to assess the extent of the viral expression and PSD-95 expression (ImageJ software).

**SUPPLEMENTARY DATA**


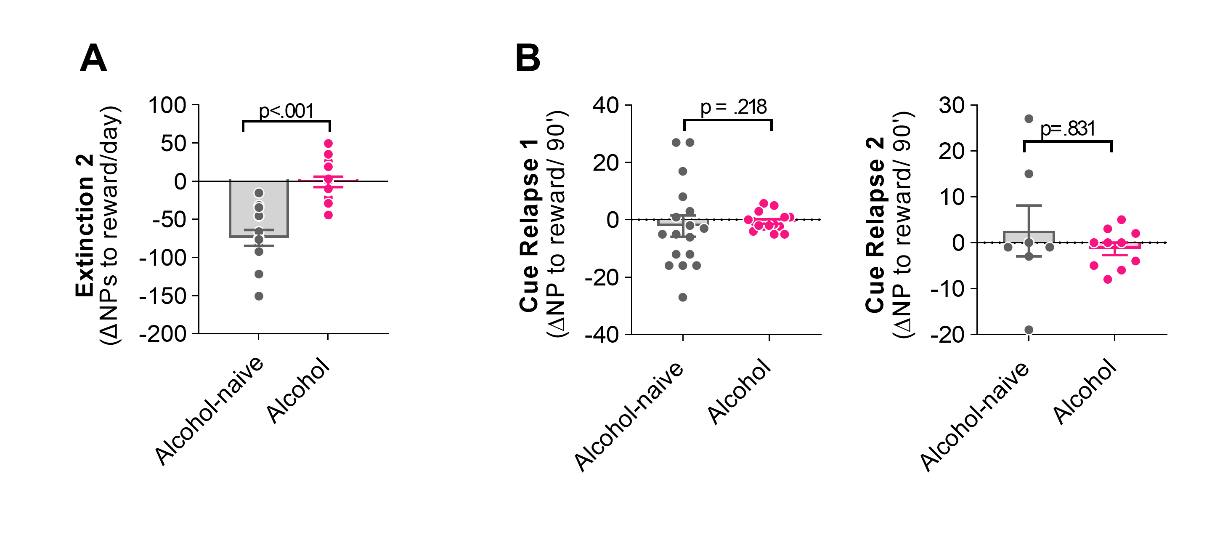


**Figure S1. The activity of the alcohol-naive and alcohol groups during second extinction and cue relapse.**

**(A)** Alcohol mice sought more for alcohol during W (t-test: t(30) = 0.391, p = 0.0004, An, n=16; A, n=16), **(B)** but not during 90-minute cue relapse 2, (CR 2: Mann-Whitney U = 32.50, p = 0.831; An, n=8; A, n=8) or the first 90 minutes of cue relapse 1, as compared to alcohol-naive animals (CR 1: Mann-Whitney U = 94.50, p = 0.218; An, n=17; A, n=16).


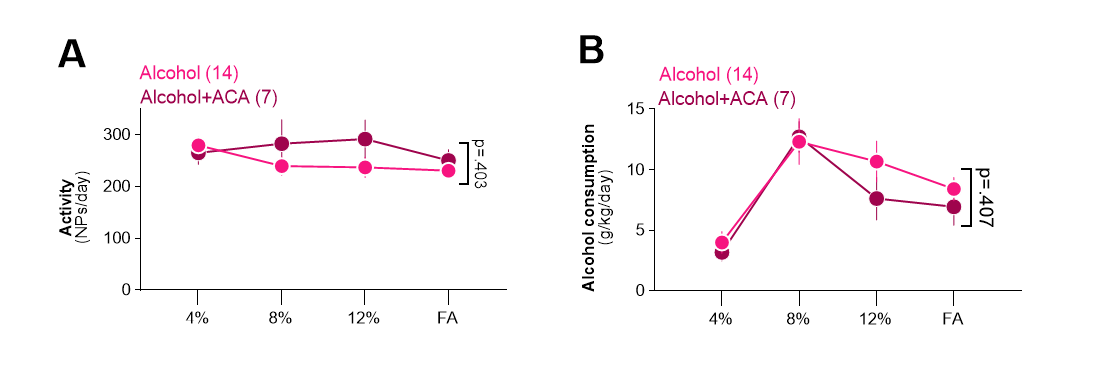


**Figure S2. Mice activity and alcohol consumption before acamprosate (ACA) treatment.**

The animals were trained in IntelliCages to drink alcohol for 57 days and next were randomly assigned to two experimental groups. Next, while still having access to alcohol, one group of mice drank ACA (250 mg/kg/day), and the rest continued alcohol consumption without any treatment.

**(A)** There was no difference between experimental groups (Alcohol vs. Alcohol+ACA) in activity (repeated measure ANOVA, ACA: F_(1, 54)_ = 0.12, p = 0.735) and **(B)** alcohol consumption before ACA treatment (ACA: F_(1, 43)_ = 0.047, p = 0.830).


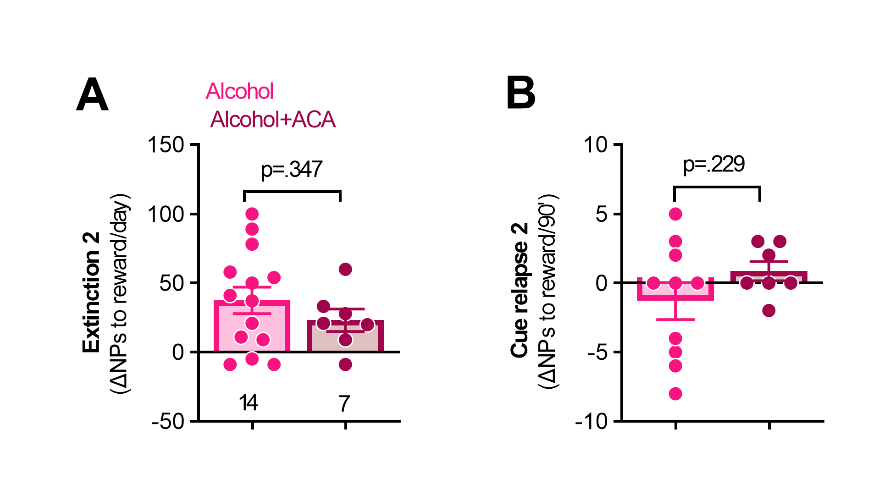


**Figure S3. The activity of the alcohol groups during extinction 2 and cue relapse 2.**

**(A)** ACA treatment did not affect alcohol seeking during extinction 2 (unpaired t-test: t(31) = 1.136, p = 0.347; A: n=16; A+ACA: n=18) and **(B)** cue relapse 2 (unpaired t-test: t(26) = 0.911, p = 0.229).


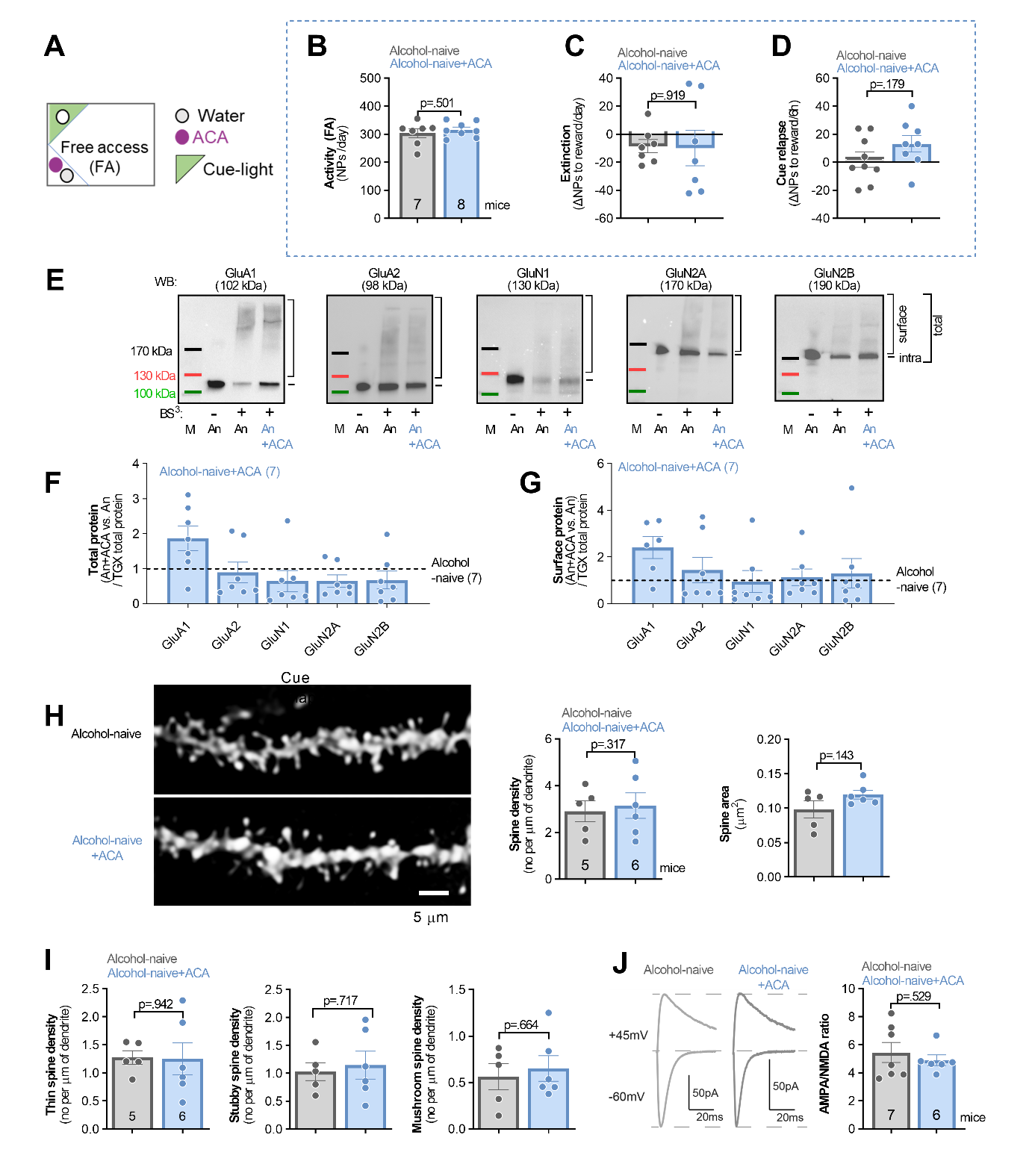


**Figure S4. ACA does not affect behavior, dDG dendritic spines and synaptic strength in alcohol-naive mice.**

**(A)** Cage setup. Mice were trained to drink water (Alcohol-naive, An) in the IntelliCages (day 1-57), and they were treated with ACA (250 mg/kg/day) (day 58-93). They underwent “alcohol-naive” W and CR during ACA treatment, and were sacrificed during W (n=7) or CR (n=7).

**(B-D)** Behavioral effects of ACA treatment of the alcohol-naive mice. Summary of data showing: **(B)** mice activity (t(13) = 0.677, p = 0.501), **(C)** alcohol seeking during “alcohol-naive” W (t(13) = 0.104, p = 0.919), and **(D)** cue light exposure (t(13) = 1.41, p = 0.179).

**(E-G)** Analysis of dDG AMPAR and NMDAR during CR after ACA treatment. **(F)** Exemplary western blots showing AMPAR and NMDAR subunits. Alcohol-naive (An) samples without BS^3^ (BS^3^-) and samples with BS^3^ are shown. M, molecular weight marker. **(F)** ACA treatment had no effect on total protein expression (GluA1: q = 0.559; GluA2: q = 0.844; GuN1: q = 0.694; GluN2A: q = 0.559; GluN2B: q = 0.692). **(G)** ACA treatment had no effect on the cell surface protein expression (GluA1: q = 0.118; GluA2: q = 0.926; GuN1: q = 0.986; GluN2A: q = 0.926; GluN2B: q = 0.926). Two-way RM ANOVA with two-stage linear step-up procedure of Benjamini, Krieger and Yekutieli for multiple comparisons, *p < 0.05.

**(H-I)** The analysis of dDG dendritic spines during CR after ACA treatment. **(H)** Representative microphotographs of dDG dendrites and summary of data showing: density (t(9) = 0.492, p = 0.317) and area of dendritic spines (t(9) = 1.605, p = 0.143) in dDG, as well as density of **(I)** thin (t(9) = 0.73, p = 0.942), stubby (t(9) = 0.373, p = 0.717) and mushroom spines (t(9) = 0.449, p = 0.664).

**(J)** Electrophysiological analysis of the dDG granule cells during CR after ACA treatment. Representative averaged EPSCs elicited by stimulations at +45 mV (top) and −60 mV (bottom) are shown. AMPA/NMDA ratio was calculated for the alcohol-naive ACA-treated and non-treated mice sacrificed during CR (t(11) = 0.649, p = 0.529).

Numbers of animals in the experimental groups are shown on the graphs (each dot represents one animal).


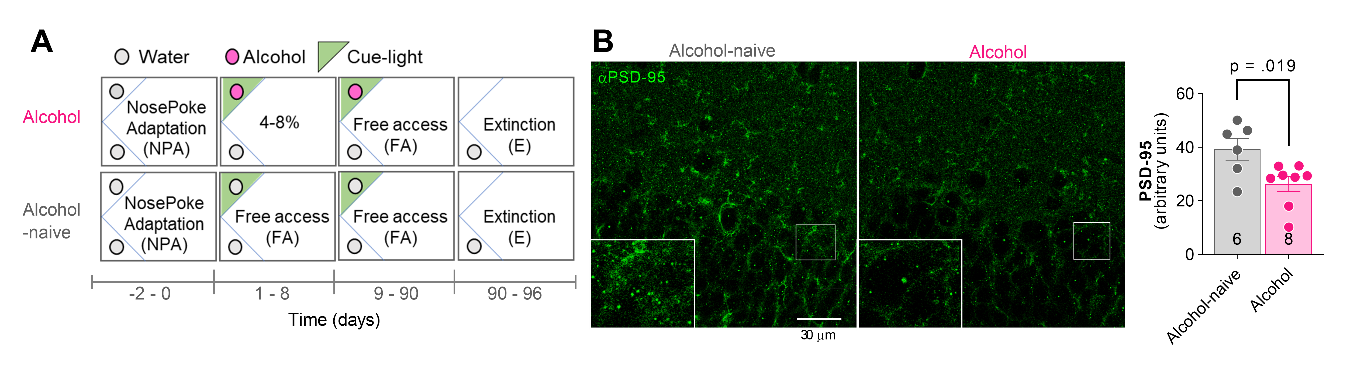


**Figure S5. PSD-95 protein levels are decreased in dDG during alcohol extinction.**

C57BL/6J female mice underwent training in the IntelliCages and were sacrificed after 6-day alcohol W (day 96). **(A)** Experimental timeline and cage setups during alcohol-related tests. The brains were sliced and stained for PSD-95 protein expression. **(B)** Exemplary microphotographs of PSD-95 immunostaining in dDG and summary of data (t-test, t(12) = 2.71, p = 0.019).


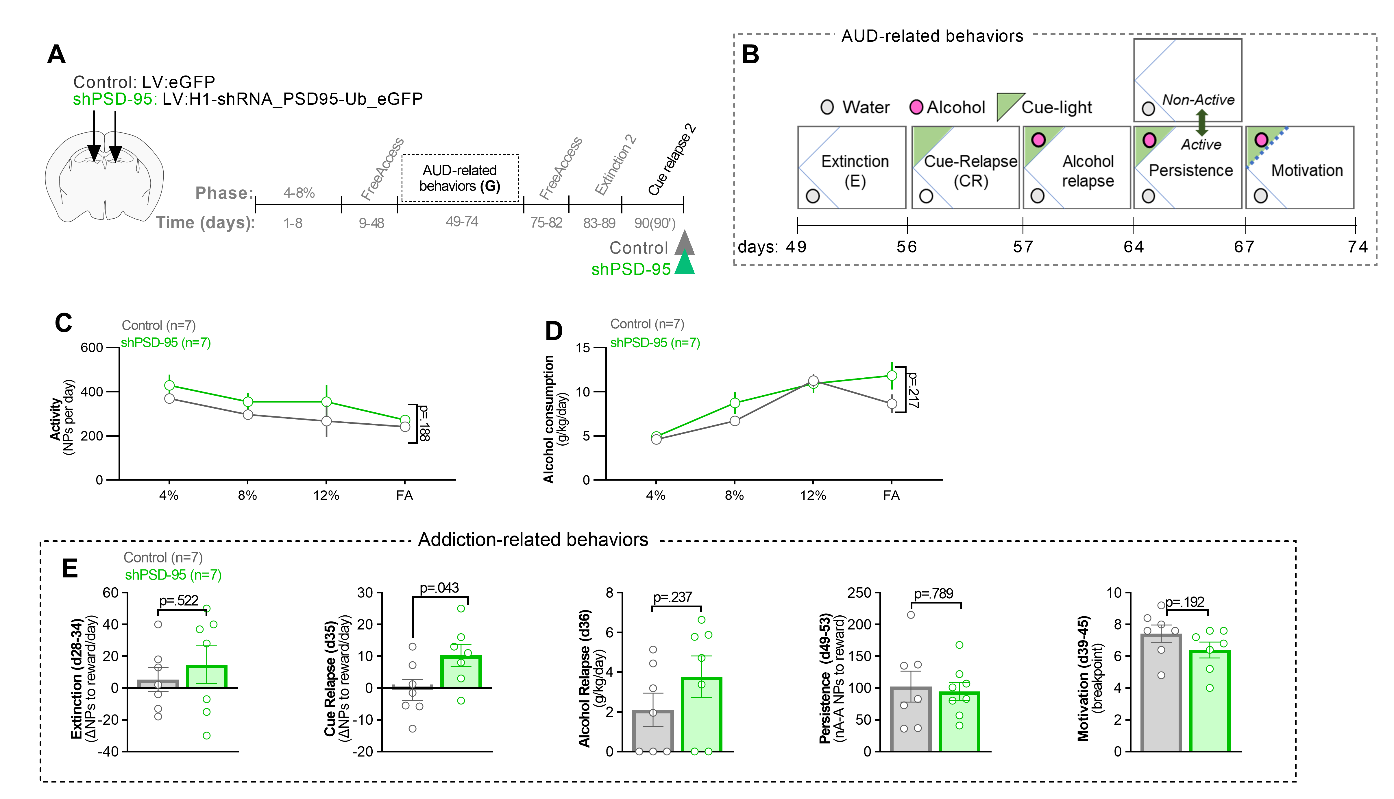


**Figure S6. Silencing PSD-95 with shRNA in dDG results in enhanced alcohol seeking during cue relapse (second cohort).**

**(A-B)** Experimental timeline. **(A)** shPSD-95 (n=8) and Control (n=7) LVs were injected into dDG. Two weeks later mice underwent training in the IntelliCages, **(B)** and alcohol-related behaviors were analyzed: alcohol seeking during extinction (E) and relapse induced by alcohol-predicting cues (CR), alcohol-drinking during relapse, motivation to drink alcohol, and persistence in alcohol seeking. Mice were sacrificed after the 90-minute CR (day 90).

**(C-D)** Summary of data showing the effects of dDG shPSD-95 on general activity. **(C)** shPSD-95 had no effect on mice activity (effect of shRNA, F_(1, 12)_ = 1.95, p = 0.188), and **(D)** alcohol consumption (F_(1, 12)_ = 1.7, p = 0.216),

**(E)** Summary of data showing the effects of dDG shPSD-95 on alcohol-related behaviors: alcohol seeking during W (t(12) = 0.660, p = 0.52), alcohol seeking during presentation of alcohol-predicting cue (t(12) = 2.26, p = 0.043); alcohol drinking during alcohol relapse (t(13) = 0.8, p = 0.237), motivation (t(13) = 0.09, p = 0.193), and persistence (t(12) = 1.26, p = 0.789).


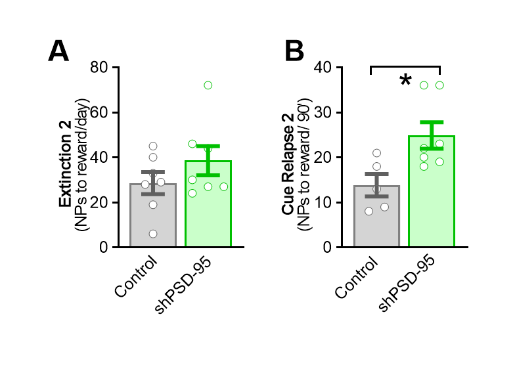


**Figure S7. The activity of Control and shPSD-95 groups during W2 and CR2.**

**(A)** shPSD-95 mice did not differ in activity during W2 (t-test: t(12) = 0.234, p = 0.819, **(B)** but showed increased CR2, as compared to the Controls (t-test: t(13) = 2.26, p = 0.042). Control (n=7) vs shPSD-95 (n=7).
